# Comparison of 22G Fork-Tip and Franseen Needles and Usefulness of Contrast-Enhanced Endoscopic Ultrasound for Diagnosis of Upper Gastrointestinal Subepithelial Lesions

**DOI:** 10.3390/diagnostics12123122

**Published:** 2022-12-11

**Authors:** Yasunobu Yamashita, Reiko Ashida, Hirofumi Yamazaki, Yuki Kawaji, Toshio Shimokawa, Takashi Tamura, Keiichi Hatamaru, Masahiro Itonaga, Masayuki Kitano

**Affiliations:** 1 Second Department of Internal Medicine, Wakayama Medical University, 811-1, Kimiidera, Wakayama, Wakayama 641-0012, Japan; 2 Clinical Study Support Center, Wakayama Medical University Hospital, Kimiidera, Wakayama 641-8510, Japan

**Keywords:** EUS-TA, subepithelial lesions, GIST, Fork-tip needle, Franseen needle, contrast-enhanced harmonic EUS

## Abstract

Endoscopic ultrasound (EUS)-guided tissue acquisition (EUS-TA) is less accurate in obtaining samples from gastrointestinal subepithelial lesions (SELs) ≤2 cm than from pancreatic cancers. The present study compared the usefulness of 22G Fork-tip and Franseen needles for EUS-TA and assessed the ability of contrast-enhanced harmonic EUS (CH-EUS) to diagnose SELs ≤2 cm. Fifty-seven patients who underwent EUS-TA for SELs ≤2 cm were evaluated. The primary endpoint was to compare the rate of acquisition of sufficient samples by these two needles. Secondary endpoints included technical success rate, adverse events, numbers of needle passes, and diagnostic ability of CH-EUS for SELs. Of the 57 included patients, 23 and 34 underwent EUS-TA with Fork-tip and Franseen needles, respectively. Technical success rates were 100% with both needles and adverse events occurred in zero (0%) and one (2.9%) patient with Fork-tip and Franseen needles, respectively. The rate of adequate sample acquisition was significantly higher using Fork-tip than Franseen needles (96% vs. 74%; *p* = 0.038). The hyper- or iso-vascular pattern on CH-EUS correlated significantly with a diagnosis of gastrointestinal stromal tumor (*p* < 0.001). EUS-TA with Fork-tip needles were superior to EUS-TA with Franseen needles in acquiring sufficient samples and CH-EUS was also useful for the diagnosis of SELs ≤2 cm.

## 1. Introduction

Gastrointestinal subepithelial lesions (SELs) are protuberant lesions or bumps covered by intact mucosa. Etiologically SELs vary from non-neoplastic lesions to true neoplasms, and their differential diagnosis ranges from benign to malignant. Gastrointestinal stromal tumors (GISTs), first described in 1983, are the most common mesenchymal tumors of the gastrointestinal tract, with a mean annual incidence of 10–15 cases per million persons. GISTs mainly affect older individuals, with a median age of 55–65 years at diagnosis [1,2,3,4]. Immunostaining tests have shown that 95%, 70%, and 30–40% of GISTs are positive for c-kit (CD117), CD 34, and smooth muscle actin (SMA), respectively, whereas fewer than 5% are positive for desmin and S-100, [5,6] making c-kit positivity diagnostic of GIST. Although 10–30% of GISTs are clinically malignant, all GISTs have some degree of malignant potential [7]. GISTs have a risk of metastatic relapse, specifically in the liver and peritoneum, after initial surgery for localized disease. Both the European Society for Medical Oncology (ESMO) and Japanese GIST guidelines have recommended surgical resection when an SEL is diagnosed as a GIST [8,9,10,11].

Endoscopic ultrasound (EUS) is the most accurate imaging method for evaluating SELs of the gastrointestinal tract [12,13,14] because it can detect the submucosal layers and the likely site of tumor origin. EUS alone, however, is not sufficient for diagnosis in many cases, such as hypoechoic and heterogeneous lesions of the submucosa and muscularis propria. Vascular assessment of lesions by contrast-enhanced harmonic EUS (CH-EUS) may be helpful in the differential diagnosis of SELs. Moreover, CH-EUS is advantageous for patients who have contraindications to magnetic resonance imaging (MRI) and CT contrast agents, such as patients with renal failure or allergy to contrast agents [15]. CH-EUS also allows for dynamic and repeat examinations, as it does not expose the patient to ionizing radiation. CH-EUS has also been reported useful for the diagnosis of SELs [16]. CH-EUS was shown to have a sensitivity of 84.5%, a specificity of 73.3%, and an accuracy of 82.2% for the diagnosis of GISTs, defined as lesions showing hyper- or iso-enhancement on CH-EUS [16].

Another option for differential diagnosis of SELs is histopathological assessment. Although histological evaluation is required for diagnosis of SELs, specimens cannot usually be obtained by conventional endoscopic biopsy methods because many of these tumors are located in deeper layers of gastrointestinal walls. Standard biopsy forceps and jumbo biopsy forceps (bite-on-bite technique) have low diagnostic yield [17,18]. Moreover, the diagnostic rate for SELs was only 38% even for two to eight bites obtained with standard-sized biopsy forceps using the bite-on-bite technique [18].

EUS-guided tissue acquisition (EUS-TA) is a minimally invasive diagnostic method consisting of two modalities: EUS-guided fine needle aspiration (EUS-FNA) and EUS-guided fine needle biopsy (EUS-FNB). EUS-TA has been used extensively to obtain samples for histopathological diagnosis of abdominal tumors, particularly pancreatic lesions [19,20]. The average diagnostic accuracies of EUS-FNA for gastrointestinal SELs have been found to range from 60 to 80% [21]. This method, however, is less accurate in the diagnosis of SELs ≤2 cm [22,23]. Moreover, EUS-FNA acquires cytological specimens, making it difficult to obtain histological architecture, and perform immunohistochemical analysis and molecular profiling. By contrast, EUS-FNB, first reported in the early 2000s, acquires tissue specimens rather than aspiration-based cytological specimens.

Novel needles were developed to acquire tissue cores in EUS-FNB. For example, 19–25 G reverse bevel needles (ProCore^TM^; Wilson-Cook Medical Inc., Winston-Salem, NC, USA) provide two cutting surfaces, a tip and a reverse bevel, enabling the preservation of histological architecture. Subsequent next-generation needles, such as Fork-tip (SharkCore^TM^; Medtronic Inc., Sunnyvale, CA, USA) and Franseen (Acquire^TM^; Boston Scientific, Malborough, MA, USA) needles, which were designed specifically to collect sufficient tissue specimens, may achieve even higher diagnostic accuracy [24,25]. Although EUS-FNB has been reported as superior to EUS-FNA for the diagnosis of SELs [26,27], the diagnostic accuracy of different types of EUS-FNB needles for SELs has not been compared. The present study therefore evaluated the utility of EUS-FNB needles and CH-EUS for SELs ≤ 2 cm and compared the diagnostic accuracies of Fork-tip and Franseen needles for these lesions.

## 2. Materials and Methods

### 2.1. Study Design

This retrospective observational study was performed at Wakayama Medical University Hospital. The study was approved by the ethics committee of Wakayama Medical University (No. 3725) and was performed in accordance with the ethical standards formulated in the 1964 Declaration of Helsinki. The first 34 patients underwent EUS-TA with 22G Franseen needles between May 2017 and October 2021, whereas 23 later patients underwent EUS-TA with 22G Fork-tip needles between October 2021 and November 2022. The primary endpoint was to compare the rate of acquisition of sufficient samples for histological evaluation by these two needles, defined as the ability to perform immunostaining on tissue samples obtained by EUS-TA. The secondary endpoints included technical success rates, adverse events, and numbers of needle passes. The utility of CH-EUS for diagnosis of SELs was also evaluated. Technical success was defined as needle penetration into the SELs.

### 2.2. Eligibility Criteria

Patients were included if they (i) were aged ≥20 years; (ii) had SEL ≤2 cm on diagnostic imaging; (iii) had a performance status ≤2; (iv) underwent CH-EUS; and (v) required histological evaluation with 22G Franseen needles (before October 2021) or 22G Fork-tip needles (after October 2021) for determination of treatment. Patients were excluded if they (i) had a bleeding tendency, defined as an international normalized ratio of the prothrombin time >1.5 or a platelet count <50,000 cells/μL; (ii) had cystic lesions; (iii) had expected difficulty of endoscope insertion; (iv) had a serious dysfunction in other organs; or (v) were otherwise judged by the investigator to be ineligible for inclusion.

### 2.3. EUS-FNB Needles (Figure 1)

Franseen needles.

Franseen needles are made of cobalt–chromium and have a crown-shaped tip with three symmetric prongs.

**Figure 1:**
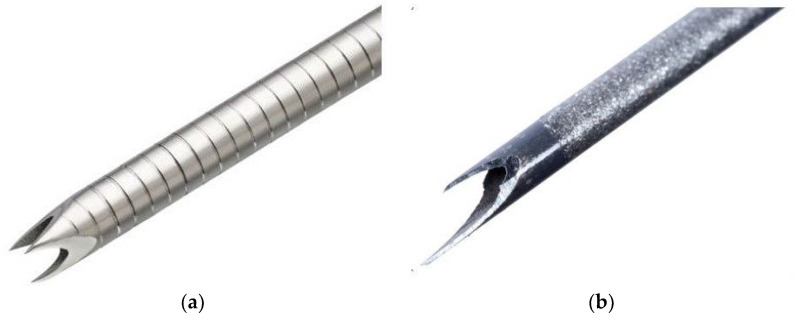
Photographs of (**a**) a 22G Franseen needle (Acquire^TM^) and (**b**) a 22G Fork-tip needle (Shark Core^TM^). (**a**) Franseen needles are made of cobalt–chromium and have a crown-shaped tip with three symmetric prongs. (**b**) Fork-tip needles are made of stainless steel and contain a nitinol stylet. The device has a multifaceted opposite bevel tip incorporating two sharp prongs of different lengths.

Fork-tip needles.

Fork-tip needles are made of stainless steel and contain a nitinol stylet. The device has a multifaceted opposite bevel tip incorporating two sharp prongs of different lengths.

### 2.4. Procedures

EUS procedures were performed using electronic convex-type echoendoscopes (GF-UCT260; Olympus, Tokyo, Japan) with an ultrasound processor (ALOKA ProSound SSD α-10; Aloka Co., Ltd., Tokyo, Japan; ARIETTA 850; FUJIFILM Healthcare, Tokyo, Japan). All procedures were performed by experienced operators who had performed at least 100 EUS-TA procedures. CH-EUS was performed using the extended pure harmonic detection method with the mechanical index set at 0.25. EUS was performed in the left lateral position under diazepam-induced sedation with heart rate monitoring. CH-EUS was performed using Sonazoid^®^ (GE Healthcare Pharm, Tokyo, Japan), a second-generation ultrasonography contrast agent composed of perfluorobuthane microbubbles with a median diameter of 2–3 μm. After reconstitution with 2 mL of sterile water for injection, 0.7 mL of the agent was administered through a peripheral vein, followed by assessment of the vascular pattern. Evaluations were made on-site by two physicians, each with at least 10 years of EUS experience. GISTs were defined as lesions showing a hyper- or iso-enhancement pattern on CH-EUS. The ability of CH-EUS to diagnose SELs was assessed in patients pathologically diagnosed by EUS-TA.

For EUS-TA, Doppler mode was used to determine whether any large blood vessels crossed the planned puncture route. The stylet was retracted approximately 5 mm, and a puncture was made. While applying negative pressure using a 10 mL syringe and monitoring the puncture needle under ultrasound guidance in real time, the needle was moved back and forth about 20 times.

During the punctures, the EUS-TA procedure was deemed complete when the operator determined that sufficient specimen had been collected using rapid onsite evaluation (ROSE). With ROSE, adequacy of aspiration specimens was ensured with a microscope in the endoscopy suite. If the aspirate revealed to contain inadequate sample, the area of puncture was changed and EUS-TA was continued to gain adequate samples. However, EUS-TA was finished at the discretion of the operator if the specimen was inappropriate for 3 or more times

### 2.5. Pathological Diagnosis

Both the EUS-TA and surgically resected tissue specimens were fixed in 10% formalin, and tissue blocks were embedded in paraffin. Samples were analyzed histopathologically by staining with hematoxylin and eosin (HE). Representative histologic sections of each tumor were subsequently immunostained using commercially available antibodies against c-kit (CD117), CD34, S-100, and SMA. GIST was diagnosed when pathologic examination showed spindle or epithelioid cells stained positively with antibody to c-kit. The risk classification of malignancy of GISTs was performed according to the modified Fletcher classification system for surgical resection [28].

### 2.6. Final Diagnosis

The final diagnosis of patients who underwent surgical resection was based on the results of surgical pathology. Patients who did not undergo surgical resection were followed up by EUS or endogastroduodenoscopy for at least 5 months, during which lesion size was measured. Patients were diagnosed with benign disease (non-GIST) if they had a nonresected mass pathologically diagnosed by EUS-TA that did not display features of malignancy during imaging follow-up. SEL with c-kit positive by EUS-TA was diagnosed as GIST.

### 2.7. Statistical Analysis

Numerical outcomes were evaluated using Student’s t-test, and qualitative outcomes were evaluated using Fisher’s exact tests. The numbers of needle passes and mass sizes were compared using Dunnett’s test. The diagnostic accuracies of CH-EUS for GIST were calculated from the receiver operating characteristics (ROC) curves. Areas under the ROC (AUROC) curve were defined as low (i.e., 0.5 to <0.7), moderate (i.e., 0.7 to <0.9), or high-accuracy (i.e., ≥0.9). All tests were two-sided, and *p*-values < 0.05 were considered statistically significant. All statistical analyses were performed using JMP Pro version 14 statistical software (SAS Institute Inc., Cary, NC, USA).

## 3. Results

Of the 57 patients who underwent EUS-TA, 3, 53, and 1 had esophageal, gastric, and duodenal tumors, respectively. A total of 23 patients underwent EUS-TA with Fork-tip needles and 34 with Franseen needles, with all procedures performed successfully. Evaluation of the 23 patients who underwent EUS-TA with Fork-tip needles showed that 15 were diagnosed with GISTs, including eight with very low risk, three with low risk, two with intermediate risk, and two with unknown risk GISTs; six were diagnosed with leiomyomas, one was diagnosed with a lipoma, and one was not diagnosed. Of the 34 patients who underwent EUS-TA with Franseen needles, 16 were diagnosed with GISTs, including 7 with very low risk, 6 with low risk, and 3 with intermediate risk GISTs; of the remaining 18 patients, 8 were diagnosed with leiomyomas and 1 with inflamed fibrous stromal tissue, whereas 9 were not diagnosed. The clinicodemographic characteristics of these patients are shown in Table 1. There were no significant differences in age (69 (range, 24–77) years vs. 66 (range 32–81) years, *p* = 0.59), male/female ratio (12/11 vs. 16/18, *p* = 0.79), mass size (18 (range, 7–20) mm vs. 15 (range, 10–20) mm, *p* = 0.23), location in the stomach/esophagus or duodenum (23/0 vs. 30/4, *p* = 0.15), and GIST/non-GIST ratio (10/6 vs. 16/18, *p* = 0.28) between patients who underwent EUS-TA with Fork-tip and Franseen needles. Rates of adverse events did not differ significantly in patients who underwent EUS-TA with Fork-tip and Franseen needles (mild bleeding) (0% (0/23) vs. 2.9% (1/34), *p* = 1.0). In 10 patients, the specimens obtained by EUS-TA were insufficient for diagnosis. Of the remaining 47 patients, 31 were diagnosed with GISTs, 14 with leiomyomas, and 1 each with a lipoma and inflamed fibrous stromal tissue.

Of the 57 patients with SELs ≤2 cm, 31 (54%) were diagnosed with GIST, and 29 underwent surgical resection; the remaining two patients did not undergo surgery. Five (17%) of the twenty-nine resected GISTs ≤2 cm were found to be intermediate-risk tumors (Table 1). The median numbers of Fork-tip and Franseen needle passes were similar (2.5 (range, 1–7) vs. 3 (range, 1–5), *p* = 0.86) (Table 2). The rate of adequate sample acquisition for EUS-TA was significantly higher using Fork-tip than Franseen needles (96% vs. 74%; *p* = 0.038) (Table 2).

**Figure 2:**
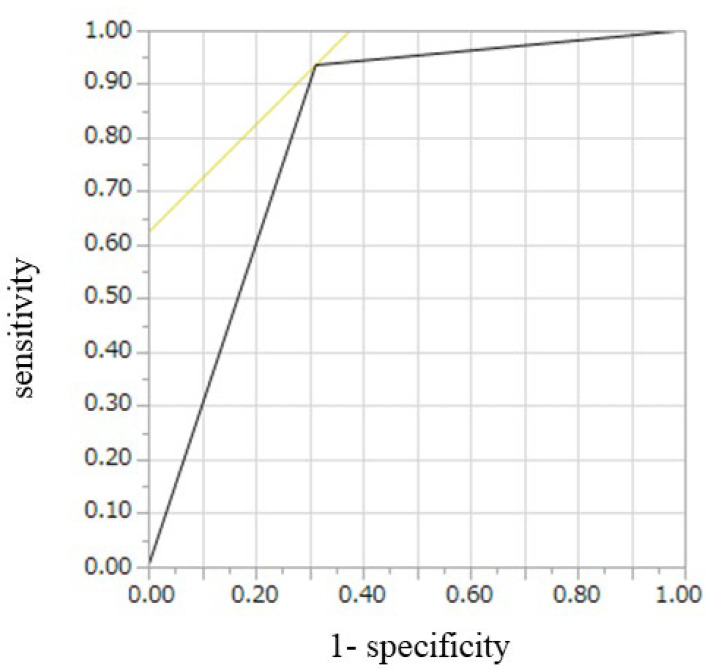
Receiver operating characteristics curve for diagnosis of GIST on CH-EUS. GIST, gastrointestinal stromal tumor; CH-EUS, contrast-enhanced harmonic endoscopic ultrasound.

CH-EUS for diagnosis of SELs was assessed in the 47 patients with final diagnoses. Of 31 GISTs, 29 had hyper- or iso-enhancement patterns and 11 of 16 non-GISTs had hypo-enhancement pattern., The sensitivity, specificity, and accuracy of CH-EUS for diagnosis of GIST were 94%, 69%, and 85%, respectively (Table 3). Hyper- or iso-vascular pattern on CH-EUS was significantly correlated with diagnosis of GIST (*p* < 0.001) (Table 3). AUROC was 0.81. CH-EUS had moderate diagnostic ability for GIST (Figure 2).

## 4. Discussion

As GISTs have some degree of malignant potential, their early diagnosis and treatment are important. An evaluation of 1765 patients with small GISTs ≤2 cm found that none had metastases [29], indicating that complete surgical resection of these tumors would be curative without the need for adjuvant therapy. In contrast, a study of patients with gastric GISTs ≤ 2 cm found that 2.5% of these tumors were of intermediate risk, and 3.6% were of high risk [30]. The present study found that 17% of GISTs ≤2 cm were of intermediate risk.

Immunostaining for the expression of proteins such as c-kit is required for confirmation of GIST because many benign SELs such as leiomyomas and schwannomas are also composed of spindle cells. Therefore, diagnosing SELs with EUS-TA is more difficult than diagnosing other tumors because accurate diagnosis of GIST requires sufficient tissue sample for immunostaining. For example, a recent meta-analysis found that the mean accuracy of diagnosing SELs was 59.9% (range, 43–91%) [31]. In particular, the diagnosis of SELs ≤ 2 cm is difficult, with a recent study reporting a diagnostic accuracy of 50% [23].

ESMO and Japanese GIST guidelines recommend surgical resection when immunostaining of SELs ≤2 cm confirms a diagnosis of GIST [8,10]. SELs were recently shown to be successfully and safely treated by endoscopic resection, as shown in a study of 972 patients with SELs ≤2 cm [32]. Minimally invasive endoscopic resection of GISTs following EUS-TA diagnosis of SELs ≤2 cm is therefore potentially curative. In the present study, 65% of the SELs ≤2 cm with final diagnosis were diagnosed as GISTs.

Several studies have compared EUS-FNB and EUS-FNA for SELs. The first randomized controlled trial (RCT), involving 22 patients with gastrointestinal SELs and using reverse bevel needles for EUS-FNB, found that the median number of needle passes required to obtain macroscopically optimal core samples were significantly lower using EUS-FNB than EUS-FNA (2 vs. 4, *p* = 0.025) [26]. EUS-FNB was superior to EUS-FNA in obtaining macroscopically (92% vs. 30%) and histologically (75% vs. 20%) optimal core samples. A second study involving 24 patients and using reverse bevel needles for EUS-FNB reported that the rate of correct diagnosis for immunostaining tended to be higher for EUS-FNB than for EUS-FNA (91.3% vs. 73.9%, *p* = 0.120) [33]. A larger RCT of 70 patients using reverse bevel needles for EUS-FNB found that EUS-FNB has significantly higher overall diagnostic accuracy than EUS-FNA (83% vs. 49%, *p* < 0.001) [27]. Next generation EUS-FNB needles, such as Franseen and Fork-tip needles, have better geometries, including higher inclination angles, than EUS-FNA needles. Therefore, the novel designs of Franseen and Fork-tip needle tips may allow for more effective capture of tissue prior to its shearing off than reverse bevel needle.

Until now, however, these two EUS-FNB needles had not been compared in SELs, although they have been compared in pancreatic masses. For example, yields of diagnostic cell blocks (96.0% vs. 92.0%, *p* = 0.32) and diagnostic adequacy at ROSE (94.0% vs. 98.0%, *p* = 0.32) did not differ significantly using Franseen and Fork-tip needles [34]. Similarly, these two needle types did not differ significantly in histologic diagnostic accuracy (85.3% vs. 90.7%, *p* = 0.45) [35]. Both needles achieved a high yield of histologic tissue samples and high diagnostic accuracy [34,35]. Moreover, a meta-analysis found no significant differences in pooled rates of diagnosis between Franseen and Fork-tip needles (92.7% vs. 92.8%, *p* = 0.98) [36].

**Figure 3:**
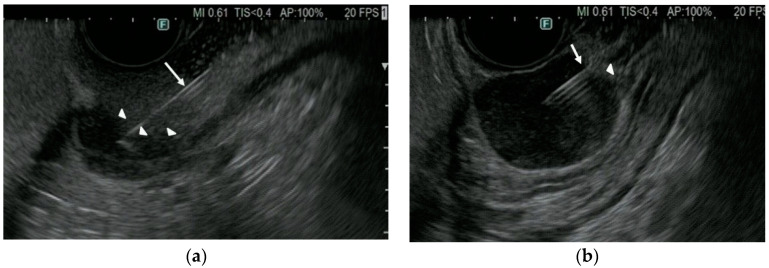
EUS imaging between Franseen (Acquire^TM^) and Fork-tip (Shark Core^TM^) needle in puncturing SEL. (**a**) Franseen needle (arrow) did not puncture SEL but pushed the surface of SEL (arrowhead). (**b**) Fork-tip needle (arrow) sharply punctured SEL (arrowhead). EUS, endoscopic ultrasound; SEL, subepithelial lesions.

By contrast, the present study found that the rate of adequate sample acquisition from SELs ≤2 cm for immunostaining was significantly higher with 22G Fork-tip (96%) than with 22G Franseen (74%) needles. SELs move more and are harder than other tumors, making it more difficult to puncture SELs ≤2 cm than other tumors with EUS-TA needles. EUS-TA of SELs therefore requires proper puncturing as well as obtaining sufficient tissue for analysis. The difference between Fork-tip and Franseen needles may be due to the number of puncture points, one and three, respectively. Resistance at the time of puncturing and penetration may therefore be lower with Fork-tip than with Franseen needles, making Fork-tip needles superior in sample acquisition (Figure 3), as well as needle mobility in tumors.

To our knowledge, this is the first study to compare EUS-TA using Fork-tip and Franseen needles for the diagnosis of SELs. These findings indicate that, in patients with SELs ≤2 cm, Fork-tip needles play an important role in the diagnosis of GISTs and in treatment decisions.

This study also demonstrated that the vascular pattern on CH-EUS correlated significantly with a diagnosis of GIST and CH-EUS is highly accurate for the diagnosis of GISTs (85%). CH-EUS showed high diagnostic ability for SELs ≤ 2 cm, similar to previous reports, indicating that CH-EUS may be diagnostically useful, regardless of SEL size. However, although EUS-TA is important for the pathological diagnosis of SELs detected on EUS, its diagnostic ability for SELs ≤2 cm is limited. Thus, in patients with small SELs, EUS-TA may not be able to acquire a sufficient sample for diagnosis. In such patients, CH-EUS should be performed to clarify the nature of the lesion, with lesions showing a hyper- or iso-enhancement pattern on CH-EUS requiring repeat EUS-TA with a Fork-tip needle or careful follow-up.

This study has several limitations. First, it included a small number of patients at a single institution, meaning that the number of patients undergoing EUS-TA with Fork-tip needles was small. Additional studies with a larger number of patients from multiple centers are required. Second, this was a retrospective study, which may have introduced bias in needle selection and the number of needle passes. Third, there was no parallel use of the two types of needles, the Fork-tip needles were used more recently, whereas the Fransen needles had been used some time ago. Therefore, it was difficult to deny the possibility that the training effect influenced the results. However, both procedures were performed by experts who had performed at least 100 EUS-TA procedures. Fourth, all tumors were not routinely resected. It is difficult to set the sample size because there is no previous report. If we could not demonstrate the usefulness of either needle, we would not proceed with the next prospective study. However, in the present study, we found the Fork-tip needle to be superior to Franseen needle. Therefore, we are currently planning a randomized prospective study to prove the superiority of the Fork-tip needle against Franseen needle on the next step.

In conclusion, the present study found that Fork-tip needles were superior to Franseen needles in acquiring sufficient sample for the EUS-TA diagnosis of SELs ≤2 cm. CH-EUS was also useful for diagnosis of SELs ≤2 cm. EUS-TA with Fork-tip needle and CH-EUS may therefore be necessary for the diagnosis of SELs ≤2 cm and for making treatment decisions.

## Figures and Tables

**Table 1:** Baseline Patient Characteristics.

	Fork-Tip Needle (n = 23)	Franseen Needle (n = 34)	*p*-Value
Age, year, median (range)	69 (24–77)	66 (32–81)	0.59
Sex, male/female	12/11	16/18	0.79
Size of mass, mm, median (range)	18 (7–20)	15 (10–20)	0.23
Location			
Esophagus	0	3	0.27
Stomach	23	30	0.14
Duodenum	0	1	1.0
Final diagnosis			
GIST	15	16	0.28
Very low risk	8	7	
Low risk	3	6	
Intermediate risk	2	3	
Unknown	2		
Non-GIST	5	9	
Leiomyoma	6	8	
Inflamed fibrous stromal tissue	0	1	
Lipoma	1	0	
Not diagnosed	1	9	

GIST, gastrointestinal stromal tumor.

**Table 2:** Comparison of Procedure-related Performances of the Fork-tip and Franseen needles.

	Fork-Tip Needle (n = 23)	Franseen Needle (n = 34)	*p*-Value
Number of needle passes	2.5 (1–7)	3 (1–5)	0.86
Technical success (puncture success)	100% (23/23)	100% (34/34)	-
Adverse event	0% (0/23)	2.9% (1/34)	1.0
Adequate sampling acquisition rate	96% (22/23)	74% (25/34)	0.038

**Table 3:** Correlation between hyper- or iso-enhancement pattern on CH-EUS and GISTs.

	Final Diagnosis	
	GIST (n = 31)	Non-GIST (n = 16)
Hyper- or iso-enhancement pattern on CH-EUS	29	5
Hypo-enhancement pattern on CH-EUS	2	11

*p* < 0.001; GIST, gastrointestinal stromal tumor; CH-EUS, contrast-enhanced harmonic endoscopic ultrasound.
